# Older adult immigrants’ experiences of being hospitalized: a qualitative study

**DOI:** 10.1186/s12913-024-11848-6

**Published:** 2024-11-12

**Authors:** Lisbeth Alnes Vestgarden, Elisabeth Dahlborg, Jeanne Strunck, Elin Margrethe Aasen

**Affiliations:** 1 Department of Health Sciences in Aalesund, Faculty of Medicine and Health Sciences, Norwegian University of Science and Technology (NTNU), Box 1517, Aalesund, N-6025 Norway; 2 Department of Health Sciences, University West, Trollhättan, 46132 Sweden; 3 Department of Culture and Learning, Faculty of Social Sciences and Humanities, Aalborg University, Kroghstraede 3, 4-23, Aalborg, 9220 Denmark

**Keywords:** Older adult immigrants, Patient, Hospitalized, Experiences, Qualitative research

## Abstract

**Background:**

Access to equal health services is a key issue in most European countries. In the coming years, immigrants will constitute an increasing proportion of older adults in Europe, and their need for healthcare services will likely increase. Healthcare services must prepare for such encounters to make them equitable. Older immigrants’ hospitalization experiences require elucidation. Their patient experiences can provide important knowledge when the healthcare system is working toward equal and equitable healthcare services.

**Methods:**

This study employed an exploratory qualitative design. Data were collected through narrative interviews with a purposive sample of eight older adult immigrants, aged 61–79 years. Patient narratives were analyzed using thematic analysis with a reflexive approach, as outlined by Braun and Clarke.

**Results:**

The analysis created three themes that shed light on older adult immigrants’ experiences as hospital patients. The themes conveyed experiences related to challenging involvement and interaction, notions of what an ideal patient should be like, and participants not feeling valued as a person.

**Conclusion:**

The findings indicate that communication between healthcare professionals and older adult immigrant patients is deficient: older immigrants in this study did not make their voices heard nor were they invited to participate by healthcare professionals. This contributes to limited involvement in assessment, treatment, and care. The older immigrants felt that they were not valued nor met as unique individuals. The findings indicate that health policy goals regarding patient participation and person-centered care are not met when older immigrants are patients. Consequently, the experiences of older adult immigrants in this study indicate that equal health services are at risk.

**Supplementary Information:**

The online version contains supplementary material available at 10.1186/s12913-024-11848-6.

## Background

This study focused on older adult immigrants who were patients in a somatic hospital ward in Norway. The number of international immigrants in Europe has increased in recent years to approximately 87 million, 40 million of whom have non-European backgrounds. Immigrants comprise 12% of the European population and 16% of the Norwegian population [1, 2]. Several European countries, including Norway, are currently experiencing an increasing proportion of older adults in their immigrant populations [3]. Forecasts for Norway indicate that the number will increase sharply in the years to come. In 2040, there may be three times more immigrants aged 65–79 years, those aged 80 years or older are expected to increase fourfold, and most will have a background from countries outside Europe [4]. In this study, following Statistics Norway, immigrants are defined as “persons who have themselves immigrated to Norway, and who were born abroad to foreign-born parents and four foreign-born grandparents” [2].

Immigrants in Norway have the same legal rights to public healthcare services as the rest of the population, and the overarching health policy goal regulated by law is that everyone should have equal access to health services and that health services should be equitable [5, 6]. Old age leads to increased use of health and care services and more days spent in hospitals as compared to their younger counterparts [7]. The increasing number of older adult immigrants will likely mean that healthcare services and personnel will encounter more immigrants as patients. Nevertheless, older immigrants in Norway use primary and specialist healthcare to a lesser extent than the rest of the population of the same age. They use fewer general practitioners, have lower use of healthcare services in their homes, and have lower use of somatic specialist health services and hospital admissions [8]. A Norwegian study found that the risk of hospital admission was influenced by socioeconomic factors: immigrants with low education or income were less likely to be hospitalized with serious conditions than the native-born population [9].

Although immigrants have legal rights to healthcare services, this does not necessarily mean that they have real access. Immigrants worldwide face additional access barriers to health services compared to the host population [10], and despite ambitions to ensure equality, persistent inequalities exist between migrants’ and non-migrants’ access to health services in Europe [11]. Immigrants can experience access barriers at the individual, structural, and system levels [12], and these barriers are influenced by factors, such as health literacy, differences in health beliefs, and language barriers [13]. Health literacy refers to people’s ability to manage health and navigate the health system, and their ability to find, understand, judge, and apply health information [14]. Navigating the healthcare system can be challenging for immigrants, as formal information is rarely available in languages ​​other than Norwegian and English [15]; the inability to speak the official language of the host country is therefore a barrier both at the individual- and system-level [16]. In Norway, immigrants are covered by the new Interpreting Act of 2021, which requires public services to use qualified interpreters if the interlocutors cannot communicate in a common language. However, challenges and underuse are reported, partly due to a shortage of qualified interpreters and their unavailability when needed [17].

Further, experiences of discrimination and racism can negatively affect access to healthcare and lead to inequalities in care [18–20]. Access to healthcare services is also influenced by the interplay of macro- and micro-contexts, such as when an ongoing negative public discourse at the macro level spills over and influences the interaction between patients and healthcare personnel at the micro level [16].

Age is a final barrier. Whether patients are immigrants or non-immigrants, age can reinforce barriers because individuals are stereotyped based solely on their age. According to the World Health Organization [21], ageism can negatively affect the quality of healthcare that older adults receive.

### Patients’ changing roles

The patient’s role has changed over the past decades, including in the Nordic region, resulting in the expectation that patients should be active participants and more involved than before in the assessments, treatment, and healthcare they receive [22]. The Shanghai Declaration highlights the importance of health literacy, enabling individuals to participate in health decisions [23]. Although patients are expected to be active and more involved than before, a Norwegian study of health policy documents at a macro level analyzed the Patients’ Rights Act and found a dominant paternalistic discourse in which patients were constructed as helpless and powerless individuals who lacked knowledge [24]. A similar Danish study found that patients were constructed as passive recipients of healthcare [25], and a Swedish study concluded that patients have a limited ability to influence their own healthcare [26].

The health policy goal of providing equal health services to the entire population of Norway is also formulated and defined at the overall macro level [27]. However, the goal of equal conditions is difficult to achieve if a patient does not have the ability, desire, or opportunity to seek healthcare services [28]. Therefore, it is highly relevant to investigate the *micro* level and explore how the individual experiences being a patient in the health service. Health services and personnel must prepare for a more diverse and heterogeneous patient population in the coming years to ensure equitable health services for all patients. There is a gap in the existing research field concerning older adult immigrants’ experiences as patients, and it is of the utmost importance to also make room for these patients’ stories. The aim of this study is therefore to explore older adult immigrants’ experiences of being a patient, in hospital.

## Methods

### Design

This study adopted a qualitative exploratory approach. Individual interviews with open-ended questions were conducted to obtain rich descriptions of older adult immigrants’ experiences as patients. The interviews were analyzed using thematic analysis with a reflexive approach, as outlined by Braun and Clarke [29], to identify, organize, interpret, and report patterns of meaning of the informants’ experiences. Reflexive thematic analysis emphasizes the researcher’s intersubjectivity and critical reflection as analytical resources, as analysis is a process of meaning-making rather than truth-seeking [30].

### Population and sample

Purposive sampling was used to obtain knowledge about the experiences of older adult immigrants [31]. Participants were selected based on specific inclusion criteria: (a) immigrants born and raised in countries outside Europe, (b) experience as a patient from a somatic ward in a Norwegian hospital, (c) aged > 60 years, (d) competence to consent and without cognitive impairment, and (e) ability to conduct the interview in Norwegian. Older adult immigrants are a heterogeneous population, and diversity in age, sex, ethnicity, reason for migration, and length of stay in Norway were desirable to get as rich material as possible.

Four hospitals in Norway were invited to participate, of which three accepted. All hospitals were located in cities with large immigrant populations. Two hospitals offer emergency care to approximately 200,000 inhabitants, and the third is a larger university hospital. One medical ward in each hospital was chosen for participation by the research leader at each hospital. The first author provided written and oral information about the project and inclusion criteria to the contact person (a nurse) in each ward. The nurses asked hospitalized patients if they wanted to participate in the project, and the contact information (telephone numbers) of the 29 individuals who agreed were shared with the first author. The first author called each person after discharge from the hospital to schedule a time and place for the interview. However, several of those who agreed to participate either did not respond to the telephone inquiry or chose to withdraw from the interview for unknown reasons.

Eight participants met the inclusion criteria and were recruited (Table 1). Participants were from Asia, Africa, the Middle East, and South America. Six men and two women were aged 61–79 years (mean age = 67 years). They had lived in Norway for 15–50 years (mean = 35 years) and all had worked in Norway. All were labor immigrants, except for one who came for family reunification.

**Table 1:** Participants

Sex	Men (*n* = 6)	Women (*n* = 2)
**Age (years)** Mean = 67	61–69 (P4, P6, P8) 70–79 (P1, P5, P7)	61–69 (P2, P3)
**Residence/years in Norway** Mean = 35	15–30 (P6, P8) 31–50 (P1, P4, P5, P7)	15–30 (P3) 31–50 (P2)

*P *Patient

### Data collection

Data were collected through eight individual interviews with open-ended questions in a narrative interview format. Storytelling is a universal human activity, one of the first things we learn as children, and a primary way in which human experiences are made meaningful [32]. A semi-structured interview guide developed for this study was used (Additional file 1). The interviews were conducted from January to August 2020, with six face-to-face interviews and two telephone interviews owing to COVID-19 and travel-related restrictions. Participants chose where the interview would take place: three in hospital meeting rooms, one in a library, one in a senior center, and one at the patient’s own home. The interviews began with the interviewer introducing herself as a former hospital nurse who is now employed as an academic nurse educator and a scientific researcher. Prior to the interviews, participants were informed about the project, both orally and in writing, by the recruitment nurses at the hospital. At the time of the interview, participants were informed again about the project and that the interview would revolve around their experiences of being a patient in the hospital. Participants were given the opportunity to ask questions before informed consent was obtained. The interviews continued with a self-introduction by the informants to map demographic data, such as age, country of birth, and length of residence in Norway.

Participants were then asked to narrate their stories. The first author, who conducted all the interviews, encouraged them to narrate what happened from the first day at the hospital until the day they left the hospital. Participants were asked to elaborate as necessary; for example, “Could you tell me more about that situation?” and “What did you say/do then?” However, as the researcher was part of the interview process, certain events may have received more attention than others.

Participants were recruited from three hospitals; however, by chance, each participant had been a patient at several different hospitals. Therefore, their narratives contained stories and experiences from eight hospitals in Norway. All interviews were audio recorded and transcribed verbatim by the first author. Each interview lasted 25–85 min, with an average length of 53 min.

### Data analysis

We followed the six-phase process for data engagement, coding, and theme development outlined by Braun and Clarke [29], albeit in a nonlinear process, moving back and forth, as the phases are not always sharply delineated.

In the first phase, familiarization with data, the first author listened to the audio recordings and re-read the interview transcripts several times to immerse herself and critically engage with the data. Immediate thoughts about what the entire dataset contained were noted as first impressions.

The second phase comprised systematic coding of the entire dataset. Coding was inductive (data-driven); however, as the researcher who created the coding was a former nurse, this influenced and shaped what was perceived and considered meaningful in the dataset. Each data item was closely read, and text segments that were considered meaningful and relevant to older adults’ experiences as patients were systematically tagged and assigned an appropriate code label. Some text segments were tagged with several different codes as they contained potentially different meanings, and both semantic (explicitly expressed) and latent (more implicit, beyond what was obviously stated) meanings were coded. Each code was specific and contained a single idea. As the dataset was reviewed several times, new codes were created or existing codes were tweaked if closely related ideas were captured. The generated codes captured specific nuances to explore the diversity and patterns of meaning in the dataset.

Further, the analytical phase shifted attention from small units of meaning (code) to larger patterns of meaning (theme). In reflexive thematic analysis, a theme is a pattern of shared meaning across cases organized around a central concept that captures multiple facets of an idea [29]. In the third phase, generating initial themes, the entire research team engaged with the codes and started a process to explore whether there were any ideas (with some similarity of meaning) across the dataset in which multiple codes could be clustered. The potential codes were clustered together as initial and tentative themes, creating patterns of meaning that were not necessarily apparent (to us) in the coding phase. We clustered and re-clustered codes in a range of ways and used the software program MindManager as a visual electronic tool to keep track of the working documents and explore the possible connections of the initial themes. These themes reflected interpretations from the research group’s perspective.

In the fourth phase, developing and reviewing themes, we moved backward and forward between the data extracts, the entire dataset, and the developed theme to check that the theme addressed our research question of how older adult immigrants experienced being a patient and that the analysis did not misrepresent the data. Preliminary themes were discussed several times, reorganized, and adjusted to make them richer and less fragmented so that each theme had its own focus and was organized around a singular, central idea.

In the fifth phase—refining, defining, and naming themes—we discussed the central organizing concept of each theme, its uniqueness and boundary, and how each theme contributed to answering the research question. We created names that captured the meaning and central concepts of each theme.

Finally, in the sixth phase, the results and report were written by the first author with critical input from the rest of the research team.

## Findings

Eight older adult immigrants were interviewed, all of whom had been patients in the medical wards of different hospitals. The analysis developed three themes that shed light on older adult immigrants’ experiences as hospital patients: (i) access to the healthcare room—a challenging interaction, (ii) adapting to be an imagined ideal patient, and (iii) not being valued as a person.

### Theme 1: access to the healthcare room—a challenging interaction

The first theme revolved around how participants experienced involvement in decisions, treatment, and care at the hospital. The theme comprised several nuances: older immigrants in this study had limited involvement in their care and treatment, they did not make their voices heard, nor were they invited to participate when assessments and decisions had to be made. However, their narratives indicated that they would like to be involved, while simultaneously avoiding involvement. For example, participants expressed that they had little involvement when it came to what, where, or how something was to happen and that they obeyed what the healthcare personnel told them:

*I am sick in such a way that I listen to them*,* they’re right. They know how to help you* (Patient 4).

Statements like this indicated a withdrawn and passive patient. Participants also expressed confidence in healthcare personnel’s assessment of choices with major consequences. One patient was asked by healthcare personnel whether a kidney transplant was desirable, to which the person replied that her wishes could not decide this because it was the healthcare personnel who knew her kidneys and body best. Knowledge was highlighted as a key point, and participants talked about how they did not have sufficient knowledge about the body, relevant diagnoses, treatment options, or how the health services worked as an institution. However, the older immigrants also stated how they had quietly disagreed with the healthcare personnel’s assessments and wondered whether other solutions might be better. Notably, this was not conveyed by them to healthcare personnel:

*Yeah*,* I have to accept it; I don’t have enough knowledge to argue with them* … *I can’t challenge a doctor’s decision* (Patient 7).

Participants largely left decisions to the healthcare personnel. However, this theme also touched on their desire for involvement. Participants who were not involved in changes or decisions expressed that they felt unprepared and apprehensive in the hospital. One person said that healthcare personnel had changed the time and place of the planned heart examination on the grounds that this was in the patient’s best interest. However, the patient was not asked and had no opportunity to express his opinion; he was only informed about the change:

*I was supposed to have an angiogram the next day*,* without me being involved at all. That was* … *it’s not abuse in any way* … *But* … *when I’m not a party to the matter*… *It’s okay that I need it*,* but I’m wise enough to judge for myself. I should be allowed to decide* (Patient 7).

This theme also included issues related to participants’ ethnicity and being an immigrant patient. One person talked about his disagreement with healthcare personnel and how he avoided speaking up because he was afraid:

*You need to talk to some older ethnic Norwegians. The older Norwegian ladies*,* they get so angry; they’re not afraid to vent their frustration. But we immigrants*,* we’re a bit more afraid of voicing our frustration because maybe we won’t get such good treatment if we say something. So*,* we prefer to keep quiet and not speak up* (Patient 5).

This statement suggests that participants felt afraid and apprehensive and were unsure of the consequences of expressing disagreement or making their voices heard. The fear that healthcare personnel could sanction immigrants more harshly than other patients could be related to a lack of trust in healthcare personnel, previous experiences of disempowerment, or other negative experiences of standing out.

### Theme 2: adapting to be an imagined ideal patient

The second theme concerned how the older immigrants had normative ideas of what an ideal patient entailed. Participants talked about how they behaved, what they did, or what they avoided doing, and the narratives indicated that they adapted to become an imagined ideal patient. A good patient was described as follows:

*I think I’m a good patient* … *I listen to what the doctor or nurse says* … *I’m one of those patients who doesn’t decide anything* (Patient 1).

Statements like this indicated that the best patients were those who left the assessments and decisions to healthcare personnel. Participants further reported that they did not ask for extra food even if they were given too small a portion and were always hungry while in the hospital. This is a way of falling into line; they understood that asking might seem bothersome, which was not in line with the notion that patients should be obedient and compliant. Concurrently, the narratives included a few examples of how patients, on certain occasions, asked healthcare personnel (what they themselves believed to be) critical questions regarding treatment, routines, or follow-up. However, participants considered those who behaved in this manner to be critical, difficult, and impatient:

*I’m a difficult patient; I’m perceived as that*,* yeah* (Patient 4). *I ask questions and ask about things* … *there probably aren’t many critical patients like me* (Patient 7).

A participant who had asked several questions assumed that the nurses talked about him like this afterwards:

*Oh my God*,* he had a lot of questions; I’ve never seen a patient like that* (Patient 5).

This suggests a perception that patients who asked questions or expressed their opinions stood out negatively and were not like the other patients.

Another aspect of the imagined ideal patient that came to light was the way participants narrated their stories; that is, their narratives were almost exclusively positive regarding hospital stay, medical treatment, and relationships with healthcare personnel.

*Being a patient was good in every way* (Patient 4). *It was great; the nurses who helped me are very good. I got food and a shower*,* and they do everything well*,* everything* (Patient 8). *Of course*,* I’m satisfied* (Patient 2). *I was 100% satisfied with the hospital and doctor and nurse and everything around me. So*,* I had no need for my wife to bring food or anything else. You get everything you need here at the hospital* (Patient 1).

The narratives also included contrasting examples; however, these references were brief and superficial. For example, participants said that they did not feel safe in the hospital, did not trust that the nurses would always come, that healthcare personnel should have assessed their symptoms a little more carefully, and that treatment should have started earlier. Several participants stated that they intended to complain, but none had done so:

*I just got a bit annoyed*,* but I can’t say anything to them. I was only going to be there for 4 or 5 days*,* and I didn’t want to cause a problem or make a fuss or anything like that* (Patient 8). *I don’t like to make a fuss or anything like that*,* so I’d rather just check myself out of the hospital and go home* (Patient 5).

When participants still emphasized that everything was fine and they were satisfied, it is likely connected to a normative notion of what being the ideal patient entails: one who conforms and adapts. The ideal patient is positive, does not make a fuss, and does not stand out.

### Theme 3: not being valued as a person

This theme expressed the fundamental need to be more than just an accommodating ideal patient. Participants expressed feeling like outsiders and being treated differently from other patients:

*I just want to be like a normal person*,* yes*,* like a normal person* (Patient 8). *And you must take into account and think about if it was me who had all those problems* … *You must think that we’re human beings just like everyone else* (Patient 3).

The statements indicated that the older immigrants had negative experiences, and the narratives included examples of participants feeling that they were not taken seriously, not feeling seen or heard, and wondering if healthcare personnel understood their situation. Being treated as a person was largely related to what nurses did or failed to do. Common courtesies, such as greeting someone, being pleasant, answering questions, or asking how they were doing, were highlighted; and the narratives indicated that when participants did not experience this, they felt invisible and not recognized as a person of value.

Further, participants wanted nurses to understand their situations and consider their individual needs. For example, they expressed that they may need extra care when they are in the hospital and said that nurses should check on them more often. However, expectations like these were neither expressed nor passed on to nurses. Participants hoped that nurses understood what they needed and did it without being prompted:

*For instance*,* when I haven’t called them for two hours*,* they should check on me. I might be dead; they won’t know*,* and I might not be able to use that alarm or whatever. They must ask*,* and they have to come* (Patient 8). *I’m not sure*… *if I ring the bell*… *if they’ll come to see what I want* (Patient 5).

These quotes highlight how patients’ trust in the nursing staff could be diminished. Participants spoke in different ways about how nervous and anxious they felt about what could happen to them. However, other participants emphasized the opposite: they felt seen and looked after because nurses often came into their rooms. However, they also stressed that it was not enough to simply pop in if the nurses did not take the patient seriously or listen to what the person had to say. One participant said that he contacted the nurse during the night because he was freezing and suspected that he had fever but was told by the nurse that everything was normal. He then called the nurse again, but was only offered an extra duvet. At both times, the patient tried to say that he felt something was wrong with his body:

*She didn’t understand my problem at all* … *So then I called again for the third time*,* and when she came*,* I said*,* “You must listen to what*,* why I’m calling you. It’s not for fun*,* I really have a problem”* (Patient 1).

The patient further said that he disliked being alone in a room with a high fever and that he was afraid that it could develop into something serious. He asked to be sent to the emergency department if the nurses in his ward could not or would not record his temperature.

Some participants provided examples of specific situations in which they felt overlooked, ignored, and powerless. One patient said that she did not get answers when she asked questions and cried when she talked about how frustrating this had been:

*When I ask*,* “Where am I going? When am I going there?” [They do not answer]. It was so difficult* (Patient 3).

Another participant had informed the healthcare personnel who were to move him into the ambulance:

*I told them*,* I’ve had heart surgery*,* and you have to do it very carefully … they didn’t talk to me … they had a problem with the stretcher and did it three or four times really hard. It hurt*,* but yeah … what can you do?* (Patient 8).

Quotes like this indicate patients who tried to speak up but who, concurrently, resigned themselves to the fact that it did not matter what they said.

## Discussion

This study explored older immigrant adults’ experiences as hospital patients. Through analysis of patients’ narratives, three themes were developed, illuminating their experiences: challenging interaction and limited involvement, adapting to be like an imagined ideal patient, and the feeling of not being valued as a person.

At first glance, one can be led to believe that the older immigrants in this study chose to be withdrawn and passive, and that limited involvement in assessment, treatment, and care was self-selected. The following discussion will, however, discuss this within the context of structural conditions, such as uneven power relationships, norms, othering, and health literacy.

Older adult immigrants in Norway are not a homogeneous group. They come from diverse backgrounds across 197 countries and vary in reasons for immigration, migration history, socioeconomic status, sociocultural background, education level, and length of residence in Norway [4, 7]. Health-related challenges also vary [7, 9], and like the general population, they may have different understandings of health and illness. Thus, their experiences are diverse. Elements of the experiences narrated by older immigrants in this study may be recognizable for patients in general, regardless of age or ethnicity. When we nevertheless choose to discuss structural conditions and the experiences of older adult immigrants in particular, it is because their immigrant background *might* be related to this. However, we are aware that this may contribute to generalization, potentially overshadowing nuances in a heterogeneous population.

Participants in this study had lived in Norway for a long time and were able to conduct the interviews in Norwegian. Therefore, these patients likely have different prerequisites for being a patient and may thus have quite different experiences to recently arrived immigrants or immigrants with limited Norwegian proficiency. Despite their ability to communicate in Norwegian, the current findings show deficiencies in interaction between health personnel and older immigrants, indicating that communication was lacking. However, long-term residence does not necessarily imply that immigrants are familiar with the healthcare system or the patient role, especially since immigrants use healthcare services less often than the rest of the population [8]. A large proportion of immigrants in Norway have good language skills [33], but this does not necessarily imply that they can express themselves adequately as patients. Patients, in general, may lack the vocabulary to describe their symptoms or medical history [34], and some illness narratives can be difficult to tell as the experiences exceed linguistic representation, referred to as “broken narratives” [35]. This challenge can be further reinforced for immigrants who have to express sensitive topics and concerns in a language other than their mother tongue. In addition, language skills can be affected by pain, fear, or acute illness [17].

The relationship between patients and healthcare personnel is asymmetrical and characterized by uneven power relationships [36], which can be reinforced when older adult immigrants are patients. The majority society, represented by healthcare personnel, possesses the most power and is in a position to define current norms and values based on what is perceived as normal. Participants in this study apparently had a notion that patients who asserted their own opinions were perceived as difficult by healthcare personnel and thus differed from the expected majority norm of what being an ideal patient entailed. A notion of normality also means that those outside the norm are marginalized, have less power, and are seen as “othered” [37]. There may be conditions at both the micro and macro levels that contribute to some patients being perceived as othered. Macro conditions, such as societal and media discourses, can influence norms and contribute to the “othering” of patients in the interactions between immigrants and healthcare personnel [16]. At the micro level, older immigrants may be othered by nurses because they do not conform to the nurses’ norms about an ideal patient [38]. Othering has an oppressive effect [39], and the negative consequences are that the person in question can be stereotypically categorized, generalized, and stigmatized [40], which makes the individual invisible, ignored, and not recognized as a unique person. Othering can thus be seen in contrast to person-centered care. Person-centered care is emphasized by the World Health Organization [22], which requires viewing each person as an individual with unique needs, wishes, and opinions.

However, the negative consequences of othering can be reduced if the person in question can “hide” or remain “invisible” [40]. Patients may adapt to resemble an imaginary ideal patient to avoid standing out, as this makes them feel othered and powerless. This may be why participants in this study quietly disagreed with the health personnel’s assessments, avoided speaking up, and obeyed. However, at the same time as they did this to avoid being othered, this also reduced their access to the healthcare room and the opportunity to participate and be involved in decisions, treatment, and care.

Access to the healthcare room and the extent to which older immigrant patients are involved can also be discussed in the context of health literacy. A larger proportion of immigrants in Norway, Europe, and the USA have lower health literacy compared to the general population [34, 41, 42]. Low health literacy is associated with reduced patient participation, in which the patient is given a rather passive role in the interactions with health personnel [43]. The ability to communicate with health personnel is part of people’s overall health literacy and is about patients being able to express how they want to handle their own health and discuss relevant options with healthcare personnel [34]. However, the findings of this study indicate that communication and interaction between healthcare professionals and older adult immigrants is deficient; older immigrants did not make their voices heard, nor were they invited to participate by healthcare professionals. This seems, however, to be about more than language skills, as all participants were able to communicate orally in Norwegian. Health literacy skills are developed from early childhood and throughout their life course [44], and although the participants in this study had lived for a long time in their new country, it is conceivable that they were influenced by the knowledge, norms, and underlying cultural values and beliefs from their country of origin. Patients in general (and perhaps immigrants in particular) may have a different conceptual understanding of what health and illness mean, and forms of expression (e.g., how to express fear, pain, or anxiety) may be culturally influenced. In addition, reactions to illness and how the “sick role” is defined and responded to can be culturally determined [44]. Older immigrants may be unsure of what is considered appropriate and normal to do as a patient; for example, how they should communicate their health problems, wishes, or questions appropriately, which may be why they remain silent and leave decisions to healthcare professionals.

Access to the healthcare room and who gets the opportunity to participate may also be determined by healthcare personnel’s normative expectations of what is considered appropriate and ideal for patients to do [43]. Patients with lower health literacy may be considered not capable of making “appropriate decisions” and are thus not given the opportunity to have a say. This entails the risk of a paternalistic approach toward patients with low health literacy. Such an attitude can be perceived as a mistrust of patients’ abilities, preventing the establishment of safe relationships in which patients dare to participate.

The findings indicate that older adult immigrants in this study want to be like imagined ideal patients, but low health literacy can contribute to a negative feeling of standing out. On the one hand, health personnel need to map patients’ varied health literacy skills to provide adapted and equal care, but at the same time, patients who are assessed and tested with low scores may feel shame and fear of being judged by healthcare personnel [43]. The patients in this study may have feared that health personnel would perceive them as ignorant patients and that stigmatizing attributes would be negatively associated with them in other situations as well. A consequence of this may therefore be that they avoided making their voices heard, refrained from asking questions, and attempted to conceal their low health literacy as they were ashamed and afraid to stand out. The World Health Organization emphasizes that focusing on health literacy is crucial in the strive for equity and equal care [45], and therefore it should not be treated solely as an individual problem but also as a societal health policy issue [43, 46].

### Strength and limitations

Credibility, transferability, dependability, and confirmability were considered to ensure trustworthiness [47]. Purposive sampling of older adult immigrants was performed to ensure credibility. While the small sample size could be a limitation, this study aimed to explore and shed light on how older adult immigrants in the Norwegian context experienced being patients. Although sample diversity was desirable, the sample comprised only two women, and most participants were labor immigrants who had lived and worked in Norway for a long time. Although the sample had little variation, this likely reflects the demographics of older adult immigrants in Norway today (of those born and raised in countries outside Europe), many of whom came to Norway in the late 60s and early 70s as labor immigrants. Other older immigrants (e.g., asylum seekers, refugees, more women, or recently arrived immigrants) likely would have provided different narratives. However, the sample was considered sufficient, as participants provided detailed narratives based on multiple hospitalizations. The shortest interview was 25 min, which could be considered a weakness; however, all participants provided detailed narratives, and we trust they conveyed what was important to them, regardless of interview length. The interviews were conducted in Norwegian, although all participants had a different mother tongue. There is a risk that meaning can be changed or lost when an interpreter is used. Therefore, an inclusion criterion was that all participants could communicate orally in Norwegian. However, it is likely that the content would have been richer if the interviewees were given the opportunity to speak their native languages.

Regarding transferability, clear descriptions of the context, data collection, analysis, and participant characteristics are provided, so that those interested are enabled to assess for themselves to whom the findings may apply to beyond the study setting. Further, the choices made during the research process are carefully described to create transparent research and strengthen dependability. The COREQ checklist (Consolidated Criteria for Reporting Qualitative Research) [48] was used to ensure that important aspects of the qualitative research were conducted and reported (Additional file 2), and Braun and Clarke’s [30] quality criteria were used to produce a compelling story about the data.

Regarding confirmability, a reflexive approach was applied. Reflexive thematic analysis emphasizes researchers’ subjectivity and critical reflection as an analytical resource, as analysis is a process of meaning-making rather than truth-seeking [30]. The research team comprised four academically employed researchers; three former clinical nurses (now academic nurse educators) and a linguist. All participants were women and non-immigrants from their respective Scandinavian countries. The researchers’ work experience with immigrant patients varied from little to no experience.

The first author who conducted the interviews was a former hospital nurse and the one in the research team who had the most experience with older adult immigrant patients. This can be both a strength and a weakness. Preconceptions and previous experience may have influenced the questions asked and thus what older immigrants narrated, and field-based knowledge may have made it easier to ask relevant follow-up questions. To manage preconceptions, a semi-structured interview guide was used, and the objective was to be open and responsive so that participants could narrate their experiences as freely as possible. Throughout the research process, the team engaged in continuous discussions to reflect on assumptions, preconceptions, and interpretations. Nevertheless, Braun and Clarke [29] emphasize that the developed themes reflect the interpretation from the researcher’s point of view, as the analysis is inevitably guided by the researcher’s preconceptions.

## Conclusion

The patients interviewed were older adult immigrants who felt that they were not valued or met as unique individuals while in the hospital. Communication between healthcare professionals and the older immigrant patients was deficient, contributing to their limited involvement in assessment, treatment, and care. Patients neither made their voices heard nor were they invited to participate by healthcare professionals. Consequently, the experiences of older adult immigrants in this study indicate that equal health services are at risk.

This study is important for nurses, nurse managers, nurse educators, and other healthcare professionals, as the findings indicate that health policy goals regarding patient participation and person-centered care are not met when older immigrants are patients. To achieve the goal of equitable health services, care must be designed according to the patients’ needs, and their voices must therefore be included. The current study has implications for future research. The findings are based on interviews with older adult immigrants. The experiences of older native patients should be investigated to explore whether ethnicity or age plays a role. Further, researchers should examine ways to foster an environment in which older adult immigrants are openly and freely allowed to communicate their opinions and wishes to healthcare personnel.

## Supplementary Information

Supplementary Material 1.

Supplementary Material 2.

## Data Availability

The dataset generated and analyzed in this study is not publicly available because of individual privacy in accordance with personal data legislation and the Norwegian Centre for Research Data but is available from the corresponding author upon reasonable request.
