# Supplementary material for: Older adult immigrants’ experiences of being hospitalized: a qualitative study
Source: BMC Health Serv Res. 2024 Nov 12;24:1381. doi: 10.1186/s12913-024-11848-6 (PMC11555928; doi:10.1186/s12913-024-11848-6)
Supplement: Supplementary file 1 — Supplementary Material 1. [file 12913_2024_11848_MOESM1_ESM.docx]

**Semi-structured interview guide**

Data for the study will be collected through narratives from older adult immigrant patients and revolve around their experiences of being hospitalized. There is limited knowledge on this subject, and the objective is therefore to be open and responsive so that the informants are allowed to narrate their experiences as freely as possible.

**Questions**

*First*, could you tell us a little about yourself, age, sex, length of residence in Norway, country of birth, and work experience?

*Furthermore*, could you tell us about the hospital stay and the encounters with the nurses? From admission until discharge (e.g. how you were received, the interaction with the nurses, how were practical situations, procedures, information, etc.).

- The interviewer will encourage narrating detailed stories from the hospital stay and ask for elaboration such as “Could you tell me more about that situation?” or “What did you say/do then?”

*Finally*, is there anything else you want to add before we end? Something important to you as an inpatient that has not been asked or talked about?
